# Brain Functional and Structural Alterations in Women With Bipolar Disorder and Suicidality

**DOI:** 10.3389/fpsyt.2021.630849

**Published:** 2021-04-22

**Authors:** Huiling Guo, Ran Zhang, Pengshuo Wang, Luheng Zhang, Zhiyang Yin, Yifan Zhang, Shengnan Wei, Miao Chang, Xiaowei Jiang, Yanqing Tang, Fei Wang

**Affiliations:** ^1^Department of Psychiatry, The First Affiliated Hospital of China Medical University, Shenyang, China; ^2^Early Intervention Unit, Department of Psychiatry, Affiliated Nanjing Brain Hospital, Nanjing Medical University, Nanjing, China; ^3^Functional Brain Imaging Institute of Nanjing Medical University, Nanjing, China; ^4^Department of Radiology, The First Affiliated Hospital of China Medical University, Shenyang, China

**Keywords:** bipolar disorder, women, suicide, amplitude of low-frequency fluctuation, gray matter volume

## Abstract

**Objective:** Suicide is the leading cause of death from bipolar disorder (BD). At least 25–50% of the patients with BD will attempt suicide, with suicide rates much higher in women patients than in men. It is crucial to explore the potential neural mechanism underlying suicidality in women with BD, which will lead to understanding and detection of suicidality and prevent death and injury from suicide.

**Methods:** Brain function and structure were measured by amplitude of low-frequency fluctuation (ALFF) and gray matter volume (GMV) in 155 women [30 women with BD and a history of suicidality, 50 women with BD without suicidality, and 75 healthy controls (HC)]. The differences in ALFF and GMV across the BD with suicidality, BD without suicidality, and HC groups were investigated.

**Results:** BD with suicidality showed significantly increased ALFF in the left and right cuneus compared with BD without suicidality and HC groups. Moreover, the GMV in the left lateral prefrontal cortex and left cuneus in BD with suicidality were significantly lower than those in BD without suicidality and HC groups, while the GMV of the right ventral prefrontal cortex was significantly decreased in both BD with and without suicidality groups.

**Conclusions:** This study, combining functional and structural neuroimaging techniques, may help to identify specific pathophysiological changes in women with BD and suicidality. Increased ALFF and less GMV in cuneus might represent the neuroimaging features of suicidality in women with BD. Investigating this potential neuromarker for suicidality in women with BD may lead to the ability to prevent suicidality.

## Introduction

Bipolar disorder (BD) is the most common underlying condition in death by suicide worldwide and has a 10-fold mortality compared with those who do not have BD (1). At least 25–50% of patients with BD have attempted suicide once over their lifetime (2, 3). Suicidality is usually defined as suicidal ideation, preparatory acts toward imminent suicidal behavior, suicide attempt, and completed suicide (4). Suicidal ideation and attempt are strongly predictive of future completed suicide and should be considered a psychiatric emergency in BD patients (5). Studies have shown that suicide attempt in BD is more common in women than in men (6, 7). Nonetheless, the pathophysiologic processes that may pre-dispose people to suicidality in BD has been poorly understood, especially in women. Identification of abnormalities in multimodal neuroimaging may help elucidate the neurobiological mechanisms for suicide risk. Thus, we undertook a comprehensive neuroimaging study in women with BD with suicidality to investigate potential changes in brain function and structure.

There is substantial evidence that functional and structural abnormalities in the brain are present in patients with BD. For example, functional magnetic resonance imaging (fMRI) findings in patients with BD suggest that those with suicidality have dysfunctional brain activity in the anterior cingulate cortex, prefrontal cortex (PFC), frontoparietal cortex, temporal–occipital cortex, cuneus, pre-cuneus, insula, and dorsal striatum (8–10). Moreover, the association between functional connectivity of the frontolimbic neural system and suicide attempt in BD has been reported in a multimodal neuroimaging study (11). In addition to these abnormal brain functional findings, structural MRI (sMRI) have shown widespread reduction in gray matter volume in BD with a history of suicidality, including anterior cingulate cortex, PFC, orbitofrontal cortex, temporal cortex, parieto–occipital cortex, corpus callosum, and basal ganglia (11–14). Furthermore, in adults with psychotic disorders (schizophrenia, schizoaffective disorder, and BD-I), patients with a history of suicide attempt exhibit significantly smaller volumes in the lingual gyrus and cuneus (15). Earlier structural studies mainly focused on major affective disorders including both unipolar and bipolar patients who had attempted suicide, and found that white matter hyperintensities were correlated with suicidality (16–19). Based on these studies, it appears that advances in neuroimaging provide unique opportunities to explore and evaluate brain function and structure in BD with suicidality.

A few studies have reported that immunobiological factors and blood gene expression biomarkers were associated with suicidality in women with mood disorders (20, 21). However, there are a few studies examining the relationship between neuroimaging markers and suicidality in women with BD in the literature. To our knowledge, only two studies of brain structure in women with BD who have attempted suicide have examined regions involved in corpus callosum (22) and prefrontal gray matter volumes (23). Multimodal neuroimaging efforts could help us understand the suicide risk of women with BD and further advance the establishment of effective suicide prevention models tailored to women with BD.

In this study, we examined whole-brain amplitude of low-frequency fluctuation (ALFF) and gray matter volume (GMV) in women with BD with a history of suicidality (including suicidal ideation and suicide attempt). Spontaneous brain activity observed through resting-state fMRI has been used to locate brain hemodynamic changes associated with diseases. ALFF analyses measure voxel-level fluctuations in the amplitude of the BOLD signal at very low frequencies (typically 0.01–0.08 Hz) (24). ALFF signal is most reliable in the gray matter (25). On the other hand, major psychiatric disorders have been examined across numerous independent sMRI studies and structural markers such as GMV are relatively stable across time and may provide trait measures of brain structure abnormalities (26). We hypothesized that women with BD who exhibit suicidality would show regional impairment of brain function and structure.

## Materials and Methods

### Participants

This study included 80 women with BD (age range = 15–50 years); 30 individuals had a history of one or more suicidal ideation or suicide attempt (BD with suicidality), and 50 had no suicidal ideation or suicide attempt (BD without suicidality). All BD patients were recruited from inpatient and outpatient services at Shenyang Mental Health Center, and the Department of Psychiatry, First Affiliated Hospital of China Medical University, Shenyang, China. The presence or absence of Diagnostic and Statistical Manual of Mental Disorders, Fourth Edition (DSM-IV) Axis I diagnosis and mood state at scanning were confirmed with the Structured Clinical Interview for DSM-IV for participants ≥18 years, and the Schedule for Affective Disorders and Schizophrenia for School-Age Children was used for participants <18 years. Exclusion criteria were (a) substance or alcohol abuse/dependence, (b) concomitant major medical disorder, (c) history of head trauma with loss of consciousness for ≥5 min or any neurologic disorder, and (d) any MRI contraindication.

For the healthy controls (HC), 75 participants were recruited through advertisement with an initial screening *via* telephone interview. HC participants had no current or lifetime DSM-IV Axis I diagnosis or history of a psychotic, mood, or other DSM-IV Axis I disorder in first-degree relatives.

Written informed consent was obtained from participants ≥18 years or parents or legal guardians of minors for participants <18 years after they were given a detailed study description. The study was approved by the institutional review board of China Medical University.

### Clinical Data

Suicidality, for this study, was defined as suicidal ideation and/or suicide attempt. Suicidal ideation was defined as thinking about ending one's own life. The suicidal ideation severity was assessed using The Beck Scale for Suicidal Ideation (27). A suicide attempt was evaluated by a trained psychiatrist to assess the lifetime suicide history by reviewing the individual's medical records. We obtained symptom measures using the 17-item Hamilton Depression Rating Scale (HAMD-17 total), The Hamilton Anxiety Rating Scale (HAMA), and the Young Mania Rating Scale (YMRS).

### Magnetic Resonance Imaging Data Acquisition and Processing

fMRI and sMRI data were acquired in a GE Signa HD 3.0T scanner with a standard eight-channel head coil at the First Affiliated Hospital of China Medical University, Shenyang, China. Functional images were collected with a gradient echo-planar imaging (EPI) sequence. The parameters were as follows: TR = 2,000 ms, TE = 30 ms, flip angle = 90°, field of view (FOV) = 240 × 240 mm^2^, and matrix = 64 × 64. Thirty-five axial slices were collected with 3-mm thickness without gap. Three-dimensional, high-resolution, T1-weighted images were collected using a 3-D fast spoiled gradient-echo (FSPGR) sequence with the following parameters: TR/TE = 7.1/3.2 ms, image matrix = 240 × 240, FOV = 240 × 240 mm^2^, 176 contiguous slices of 1 mm without gap. Participants were instructed to rest with their eyes closed but remain awake during scanning.

### Functional Magnetic Resonance Imaging Data Processing

Statistical Parametric Mapping 8 (SPM8, http://www.fil.ion.ucl.ac.uk/spm) and Data Processing Assistant for R-fMRI (DPARSF, http://www.restfmri.net/forum/DPARSF) toolkits were used for functional brain images. For each participant, the first 10 images of scanned data were deleted due to the initial signal's instability. The remaining images were realigned, then spatially normalized to MNI space. The fMRI data were resampled to voxels 3 × 3 × 3 mm^3^ during normalization and smoothed at 6-mm full width at half maximum. ALFF values were calculated in each frequency band (0.01–0.08 Hz) using linear detrending. Time-series linear detrending and temporal band-pass filtering (0.01–0.08 Hz) were performed in these bands to remove low-frequency drifts and high-frequency physiological noise. ALFF at each voxel represents the averaged square root of the power in the above frequency windows normalized by the mean within-brain ALFF value for that subject.

### Structural Magnetic Resonance Imaging Data Processing

The VBM8 toolbox (http://dbm.neuro.uni-jena.de/vbm8/) was used for structural brain image processing, which was loaded into the SPM8 software. According to the default parameters of VBM8, all images were processed with a segmentation function implemented for bias correction, spatial normalization, modulation to account for volume changes in the warping, and segmentation of the original structural images in the same model. Finally, modulated gray matter images were smoothed with an 8-mm full width at half maximum isotropic kernel. These spatially smoothed gray matter images were then used for subsequent VBM statistical analysis.

### Statistical Analysis

For primary hypothesis testing, we conducted one-way ANCOVA in SPM to assess group differences in ALFF values and GMV, with age as a covariate. We set statistical significance at a corrected *P* < 0.001 (GRF-corrected). ALFF values and GMV were extracted from each cluster in which three-group differences (BD with suicidality vs. BD without suicidality vs. HC) were detected, followed by Bonferroni correction to control for multiple comparisons (BD with suicidality vs. HC, BD without suicidality vs. HC, and BD with suicidality vs. BD without suicidality). To further identify the differences in BD with suicidality, we subdivided the group into BD with suicidal ideation and suicide attempt, and analyzed the difference in the abovementioned difference regions between the two groups. The detailed methods and results are provided as Supplementary Material.

Potential differences in age and educational level across all three groups were assessed separately by one-way analysis of variance (ANOVA) and chi-square tests. Between the BD with suicidality and BD without suicidality groups, two-sample *t*-tests and chi-square-tests were used to assess within-group differences in mediation status, HAMD-17 total, HAMA, and YMRS scores. Additionally, we performed partial correlation analyses, controlling for age, between ALFF or GMV, and symptom scores (HAMD-17 total, HAMA, and YMRS scores) in BD with suicidality group and in BD without suicidality group. Statistical significance was determined by *P* < 0.05. Continuous variables were represented as average (Mean), and standard deviation (SD); categorical variables were represented as frequency (*N*) and percentage (%).

## Results

### Demographic and Clinical Variables

We found no significant differences in age and educational level among the BD with suicidality, BD without suicidality, or HC group. The BD with suicidality and without suicidality groups did not differ in medication status, HAMD-17 total, and YMRS scores, while they differed significantly in HAMA scores (*t* = 2.69, *P* < 0.05). Detailed demographic and clinical details are presented in Table 1.

**Table 1:** Clinical and demographic characteristic of all subjects.

**Characteristic**	**BD with suicidality (*****n*** **= 30)**		**BD without suicidality (*****n*** **= 50)**		**HC (*****n*** **= 75)**		**Analysis**	
	**Mean**	**SD**	**Mean**	**SD**	**Mean**	**SD**	***t*/*F***	***P***
Age (years)	25.77	9.18	27.02	9.47	30.54	11.55	2.94	0.06
Education (years)	12.80	2.94	13.00	3.42	12.99	2.75	0.05	0.95
HAMD-17 total	14.50	10.92	10.10	9.59	-	-	1.88	0.06
HAMA	14.60	11.72	8.06	8.04	-	-	2.69	0.01
YMRS	5.83	8.96	7.28	10.24	-	-	−0.64	0.52
	***N***	**%**	***N***	**%**	***N***	**%**	***χ***^**2**^	***P***
Medication (yes)	24	80%	36	72%	-	-	0.64	0.42

*Continuous variables were represented as average (Mean), and standard deviation (SD); categorical variables were represented as frequency (N) and percentage (%). BD, bipolar disorder; HAMD-17 total, the 17-item Hamilton Depression Rating Scale; HAMA, Hamilton Anxiety Rating Scale; YMRS, Young Mania Rating Scale*.

### Imaging Differences Across the Diagnostic Group

**Figure 1:**
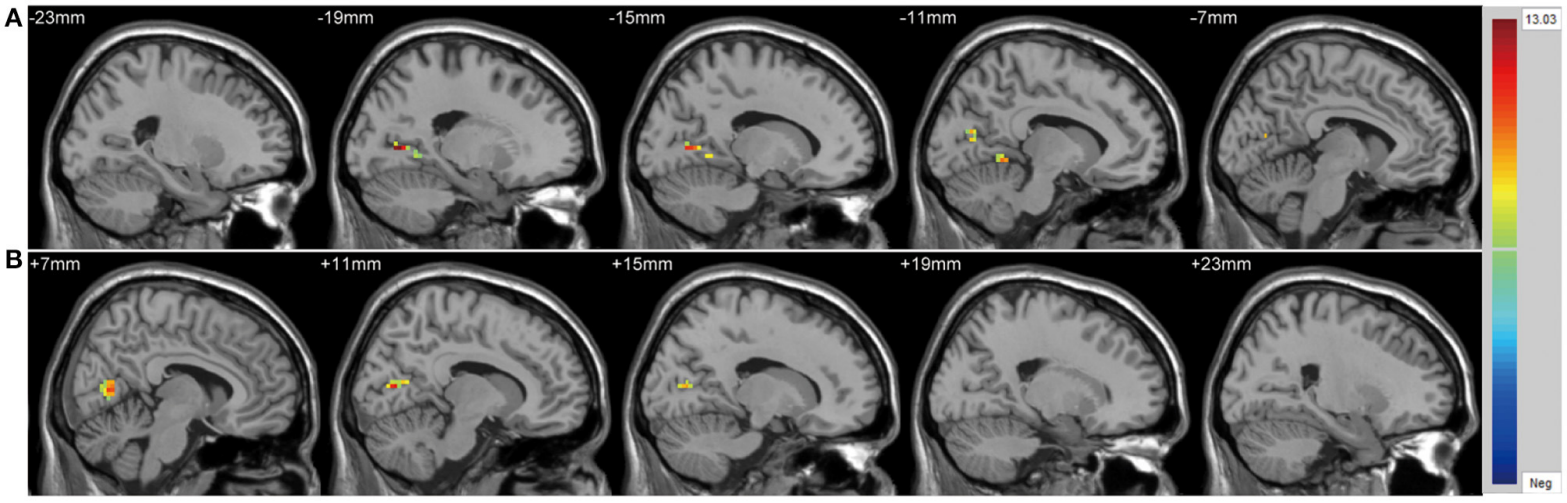
Significantly alteration of amplitude of low-frequency fluctuation values in **(A)** left cuneus and **(B)** right cuneus across the BD with suicidality, BD without suicidality, and healthy controls groups. The significance level was set to *P* < 0.001 by Gaussian random field correction. The color bar indicates the *t*-values. BD, bipolar disorder.

We found significant differences in ALFF values across the three groups in the left cuneus (*F* = 13.01, *x* = −18, *y* = −66, *z* = 3, *P*_GRF_ < 0.001, Figure 1A) and right cuneus (*F* = 13.03, *x* = 9, *y* = −66, *z* = 12, *P*_GRF_ < 0.001, Figure 1B).The BD with suicidality group demonstrated significantly increased ALFF values in the left cuneus and right cuneus compared with the BD without suicidality and HC groups (*P* < 0.05, Bonferroni corrected, **Figure 3A**); however, the BD without suicidality and HC groups did not show significant differences in the two regions (*P* > 0.05, Bonferroni corrected, **Figure 3A**).

**Figure 2:**
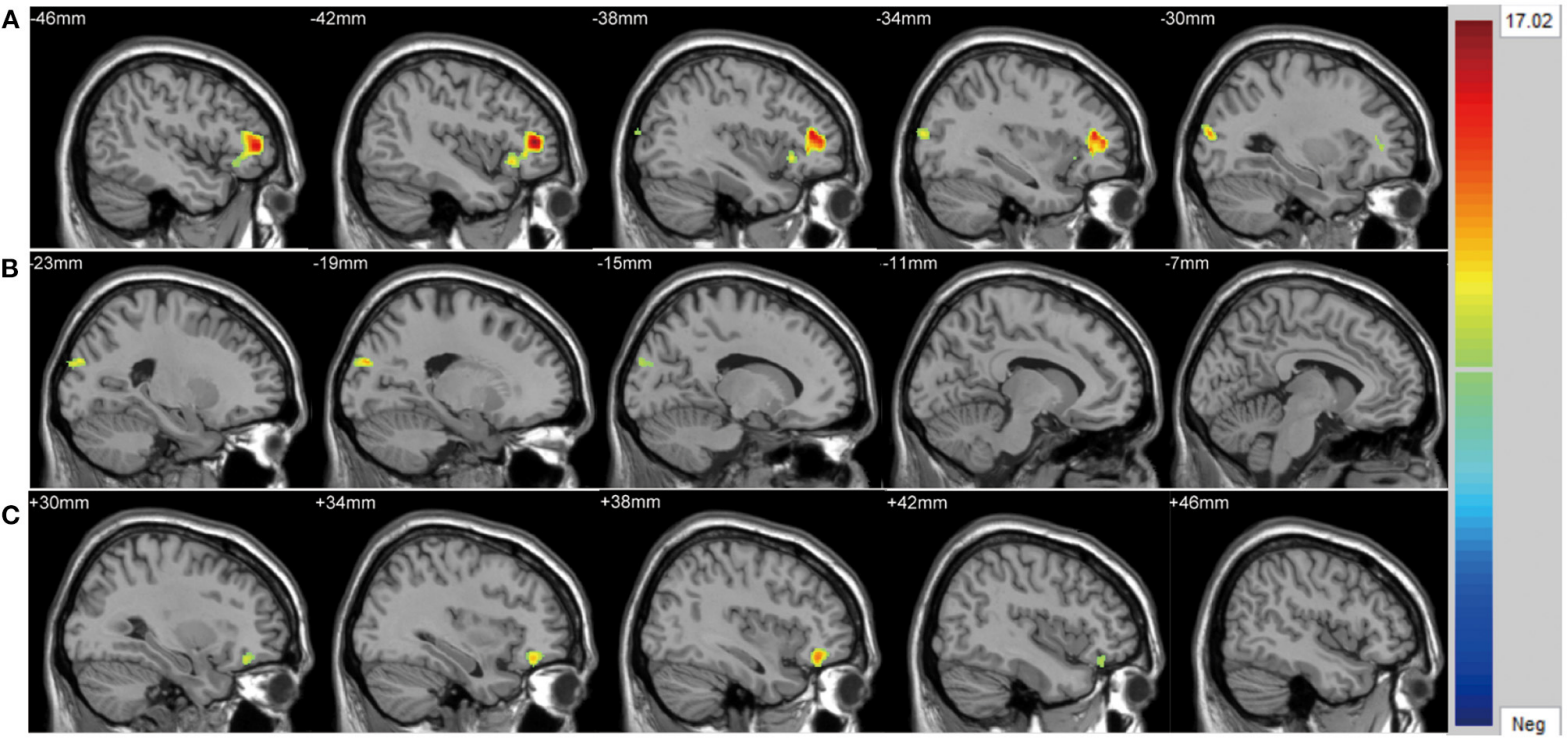
Significantly alteration of gray matter volume in **(A)** left lateral PFC, **(B)** left cuneus and **(C)** right ventral PFC across the BD with suicidality, BD without suicidality, and healthy controls groups. The significance level was set to *P* < 0.001 by Gaussian random field correction. The color bar indicates the *t*-values. BD, bipolar disorder.

**Figure 3:**
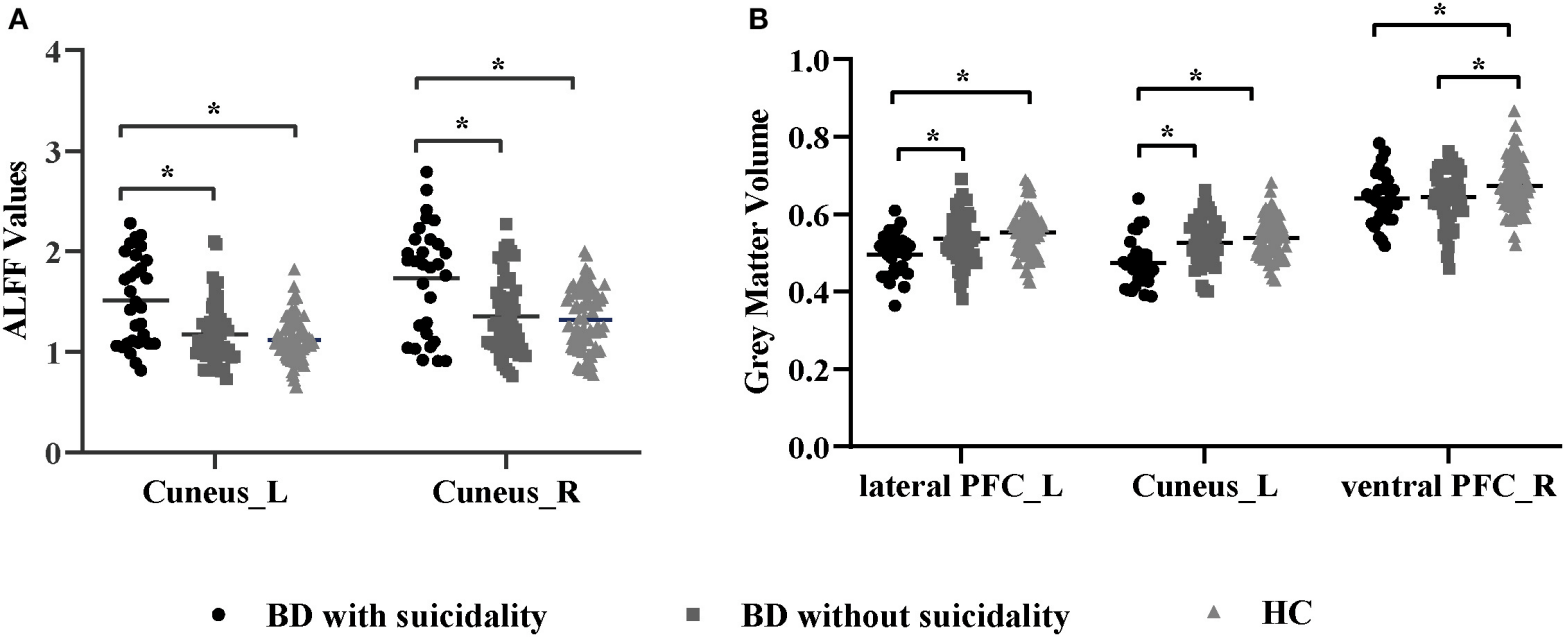
**(A)** ALFF values in regions showing significant differences among the BD with suicidality, BD without suicidality, and HC groups. **(B)** Gray matter volume in regions showing significant differences among the BD with suicidality, BD without suicidality, and HC groups. The significance level was set to *P* < 0.05 by Bonferroni corrected. *P < 0.05. ALFF, amplitude of low-frequency fluctuation; BD, bipolar disorder; HC, healthy controls; L, left; R, right; PFC, prefrontal cortex.

There were significant differences in GMV across the three groups in the left lateral PFC (*F* = 17.02, *x* = −44, *y* = 42, *z* = 6, *P*_GRF_ < 0.001, Figure 2A), left cuneus (*F* = 11.78, *x* = −30, *y* = 89, *z* = 14, *P*_GRF_ < 0.001, Figure 2B) and right ventral PFC (*F* = 12.18, *x* = 38, *y* = 36, *z* = 17, *P*_GRF_ < 0.001, Figure 2C). The BD with suicidality group demonstrated significantly lower GMV than the BD without suicidality and HC groups in the left lateral PFC and left cuneus (*P* < 0.05, Bonferroni corrected, Figure 3B); however, the BD without suicidality and HC groups did not show significant differences in the two regions (*P* > 0.05, Bonferroni corrected, Figure 3B). We found lower GMV of the right ventral PFC in the BD with suicidality and BD without suicidality groups compared with the HC group (*P* < 0.05, Bonferroni corrected, Figure 3B); however, no significant difference was found between the two BD patients' groups (*P* > 0.05, Bonferroni corrected, Figure 3B).

### Correlations Between Amplitude of Low-Frequency Fluctuation, Gray Matter Volume, and Symptom Scores

In the BD with suicidality group, no significant correlations were found between the ALFF or GMV values in the regions that showed significant differences and symptom scores (*P* > 0.05, partial correlation analysis). No significant correlations were observed in the BD without suicidality group.

## Discussion

This study was designed to analyze and compare the functional and structural alterations in the brains of women with BD with or without suicidality. Our current findings confirm and further elucidate that women in the BD with suicidality group had significantly increased ALFF in visual regions, including the left and right cuneus. These differences in ALFF were not observed in either BD without suicidality or HC groups. GMV analyses demonstrated differences among the three groups across the left lateral PFC, left cuneus, and right ventral PFC. Furthermore, BD with suicidality had a more diminished GMV in the left lateral PFC and left cuneus. However, compared with HC, the GMV of the right ventral PFC was significantly decreased in both BD with or without suicidality groups. Interestingly, the abnormality of both ALFF and GMV in the cuneus were observed in women with BD with suicidality, which indicates that the cuneus may be an important feature in the pathogenesis of suicidality in women BD patients. Of note, we found no significant correlations between ALFF or GMV and clinical symptoms, suggesting that ALFF and GMV abnormality alteration may be trait features of suicidality in women with BD, independent of the clinical symptoms' alterations. Due to the small number of subjects involved in this study, larger sample studies are needed to confirm the association between symptoms and neuroimaging parameters in women BD patients with suicidality.

The cuneus is involved in basic visual processing, receiving information from the retina (28), and receiving feedback connectivity from higher functional areas (29, 30), namely, the primary visual cortex, or visual 1 (V1). Abnormalities in the cuneus in BD patients might reflect a cognitive processing bias in subjects with BD with suicidality, suggesting the overprocessing of negative situational information. Our study was the first to combine multimodal neuroimaging techniques, such as resting-state brain function and morphology, to determine the functional activation and GMV changes in women BD patients with suicidality. In line with the present study, brain activity alteration in the cuneus has been reported in previous studies using fMRI methods. In a study with fMRI during cognitive control task performance where people with recent-onset psychotic major mood disorders (BD type I and major depressive disorder) were recruited, suicidality was associated with relatively lower activity in cuneus and pre-cuneus (9). Another fMRI study also showed that BD with suicide attempt showed abnormal brain activity in the cuneus (31). Meanwhile, an sMRI study reported that BD patients with a history of suicide attempt had significantly smaller cuneus volume (15). We also found that the left cuneus GMV was reduced in BD with suicide attempt compared with BD with suicidal ideation. However, we did not detect differences in ALFF values between the two groups, which could be due to the difference in detection sensitivity between the two methods. To sum up, our study presented new evidence to support the hypothesis that cuneus functional and structural alterations may signal risk of suicidality in women with BD. Longitudinal studies are needed to clarify the critical clinical factor that contributes to the progression of suicidal ideation to suicide attempt.

In this study, we found reduced GMV in the left lateral PFC in women with BD with suicidality, yet no significant volumetric difference in BD without suicidality. Little information is currently available about the neuroimaging change in women with BD with suicidality, with the exception of two studies revealing that suicidality in women with BD was related to the structural change of PFC (22, 23). Consistent with our findings, previous fMRI studies also demonstrated decreased dorsolateral PFC GMV in BD with suicide behavior (12, 15, 23). Additionally, the alterations in white matter integrity in ventrolateral PFC and dorsolateral PFC was associated with future suicide attempt and could represent risk factors for future attempt (32). Moreover, functional connectivity of the dorsal anterior cingulate cortex with the bilateral ventrolateral PFC and dorsolateral PFC was associated with suicidality (8). The impaired function of PFC involved in regulation of negative emotion (33) and cognitive functions (34, 35) may compromise the ability to process negative emotion and cognition, which results in hopelessness and self-destructive behavior. Our results, along with the proven psychological function of lateral PFC, demonstrates that lateral PFC damage may be associated with the pathophysiological mechanism of suicidality in women with BD. However, we could not detect significant differences of lateral PFC in the functional analyses between women with BD with/without suicidality groups, which could be the result of insufficient numbers of suicidal subjects to detect differences.

We found lower GMV in the right ventral PFC in women with BD with/without suicidality groups compared with HC, but no significant difference was seen between the two BD groups. The result of ventral PFC volumes that decreased were in line with similar findings in BD patients (36, 37). This is further confirmation that the structural alterations in ventral PFC volume did not appear to be a root cause of suicide in women with BD, but may be linked to the underlying mechanism of the disorder. Interestingly, however, another multimodal neuroimaging study of youth with BD found that the suicide attempter group had reduced white matter integrity and functional connectivity in the left ventral PFC compared with the non-attempter group (11). The aforementioned study was conducted on samples of youth, while the participants included in our study were all women, which could be responsible for the difference in the findings.

The primary strength of this study is the investigation of brain function and structure changes in women with suicidality using two MRI methods (fMRI and sMRI) in a homogenous sample. However, there are still some limitations. First, the effect of medications on the association between women with BD and neuroimaging was not considered in our analysis. Second, although a covariance analysis was carried out in the study with age as the covariate, the age range was large, and the sample included youths and middle-aged persons. Finally, we did not include the total intracranial volume (TIV) as a covariate in our study, which may affect our GMV analysis. To verify the effect of TIV, we performed the same analysis including the age and TIV as covariate, and observed the same results for regions for which women with BD with suicidality present lower GMV in the left cuneus. We did not find a significant difference in the left lateral PFC and the right ventral PFC, which was inconsistent with our original hypothesis. This demonstrates that TIV may influence PFC results independent from other factors. However, the finding of PFC in women with BD remains controversial. A study that considered TIV as a covariate has shown that suicide attempt history in women with BD was related to the GMV of PFC (23). The relatively small sample size in the current study limited the power of statistical analyses. Thus, large sample studies should be employed in the future to address the limitations of the present study and strengthen the evidence.

In summary, an integrated study of brain functional and structural neuroimaging in women with BD with suicidality suggests that abnormal function and structure in cuneus regions may be associated with suicidality. These findings provide further evidence that functional and structural abnormalities exist in suicidal women with BD by contrasting subjects with suicidal history and subjects without suicidal history within the same diagnostic category. Specifically, functional and structural abnormalities in the cuneus may represent potential neurobiomarkers of suicidality in women with BD.

## Data Availability Statement

The raw data supporting the conclusions of this article will be made available by the authors, without undue reservation.

## Ethics Statement

The studies involving human participants were reviewed and approved by First Affiliated Hospital of China Medical University. Written informed consent to participate in this study was provided by the participants' legal guardian/next of kin.

## Author Contributions

FW and YT conceptualized and supervised the study. HG and RZ participated in the design of the study, collected the data, participated in the statistical analysis, the interpretation of the data, and the drafting of the article. PW, LZ, ZY, and YZ participated in the acquisition, analysis, and interpretation of the data. SW, MC, and XJ participated in the design of the study and critically revised the manuscript for important intellectual content, and provided technical support. All authors contributed to the article and approved the submitted version.

## Conflict of Interest

The authors declare that the research was conducted in the absence of any commercial or financial relationships that could be construed as a potential conflict of interest.
