# Supplementary material for: Brain Functional and Structural Alterations in Women With Bipolar Disorder and Suicidality
Source: Front Psychiatry. 2021 Apr 22;12:630849. doi: 10.3389/fpsyt.2021.630849 (PMC8100509; doi:10.3389/fpsyt.2021.630849)
Supplement: Supplementary file 1 [file Presentation_1.pdf]

Participants

We divided BD bipolar disorder (BD) with suicidality into BD with suicidal ideation (N=10) and suicide attempt (N=20) subgroups. The differences of amplitude of low-frequency fluctuation (ALFF) and gray matter volume (GMV) between subgroups of BD with suicidal ideation and BD with suicide attempt were also explored in the regions showing significant differences among the BD with/without suicidality and healthy controls (HC) groups. A two-sample t-test was performed between the two groups.

Results

Demographic and clinical variables

We noted no significant differences in age, educational level, medication status, the 17-item Hamilton Depression Rating Scale, Hamilton Anxiety Rating Scale, and Young Mania Rating Scale score between the BD with suicidal ideation and BD with suicide attempt. Detailed demographic and clinical details are presented in Table S1.

Imaging differences across diagnostic groups

There were no significant differences in ALFF between BD with suicidal ideation and suicide attempt ($P > 0.05$, Bonferroni corrected, Figure S1). Interestingly, results revealed that BD with suicide attempt had a more diminished GMV in the left cuneus than BD with suicidal ideation ($P < 0.05$, Bonferroni corrected, Figure S1). However, there were no differences in GMV in left lateral prefrontal cortex (PFC) and right ventral PFC between the two groups ($P > 0.05$, Bonferroni corrected, Figure S1).

Table S1: Clinical and Demographic Characteristic of Patients with Suicidality

Characteristic	BD with suicidal ideation (n=10)		BD with suicide attempt (n=20)		Analysis	
	Mean	SD	Mean	SD	*t*	*P*
Age (years)	27.30	11.34	25.00	8.12	-0.64	0.53
Education (years)	13.20	3.58	12.60	2.64	-0.52	0.61
HAMD-17 total	13.80	10.52	14.85	11.38	0.24	0.81
HAMA	13.30	11.80	15.25	11.92	0.42	0.83
YMRS	4.80	2.69	6.35	2.09	-0.44	0.66
	N	%	N	%	χ^2	*P*
Medication (yes)	7	70%	17	85%	0.94	0.33

Continuous variables were represented as average (Mean), and standard deviation (SD); categorical variables were represented as frequency (N) and percentage (%). BD, bipolar disorder; HAMD-17 total, the 17-item Hamilton Depression Rating Scale; HAMA, Hamilton Anxiety Rating Scale; YMRS, Young Mania Rating Scale.

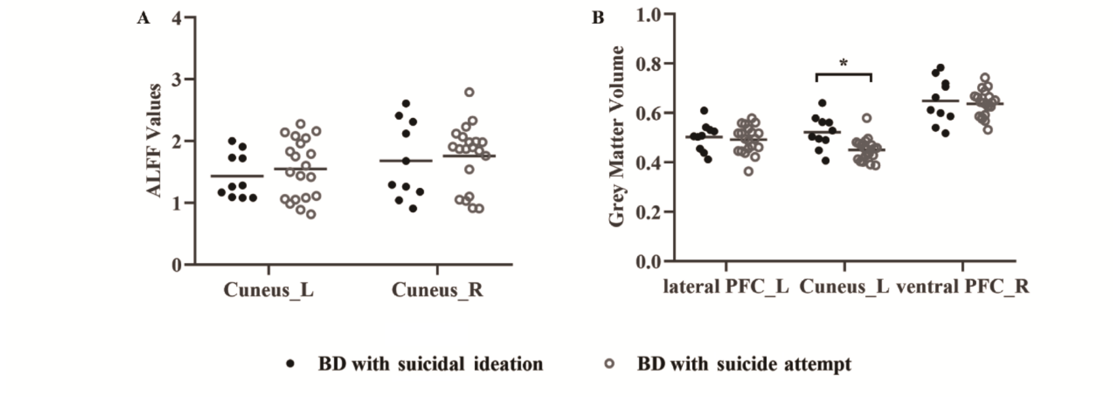

Figure S1. A. ALFF values in regions showing significant differences among the BD with suicidal ideation and BD with suicide attempt groups. B. Grey matter volume in regions showing significant differences among the BD with suicidal ideation and BD with suicide attempt groups. The significance level was set to $P < 0.05$ by Bonferroni corrected. *, $P < 0.05$. ALFF, amplitude of low-frequency fluctuation; BD, bipolar disorder; HC, healthy controls; L, left; R, right; PFC, prefrontal cortex.
